# A Systematic Review and Meta-Analysis of the Efficacy of Anti-*Toxoplasma gondii* Medicines in Humans

**DOI:** 10.1371/journal.pone.0138204

**Published:** 2015-09-22

**Authors:** Hai-Xia Wei, Shan-Shan Wei, David S. Lindsay, Hong-Juan Peng

**Affiliations:** 1 Department of Pathogen Biology, Guangdong Provincial Key Laboratory of Tropical Disease Research, School of Public Health and Tropical Medicine, Southern Medical University, Guangzhou, Guangdong, 510515, the People’s Republic of China; 2 Department of Biomedical Sciences & Pathobiology, Virginia-Maryland Regional College of Veterinary Medicine, Virginia Tech, Blacksburg, Virginia, United States of America; Institut national de la santé et de la recherche médicale - Institut Cochin, FRANCE

## Abstract

No effective drug and definitive “gold standard” treatment for *Toxoplasma gondii* (*T*. *gondii*) infection has been available so far, though some medicines have been commonly used in the treatment of *T*. *gondii* infection, such as spiramycin, azithromycin, traditional Chinese medicine (TCM), pyrimethamine- sulfadiazine (P-S), trimethoprim-sulfamethoxazole (TMP-SMX), and pyrimethamine-clindamycin (P-C). A systematic review and meta-analysis were performed to compare the efficacies of these conventional medicines in the treatment. Cohort studies for the treatment of acute *T*. *gondii* infection were searched from PubMed, Google Scholar, ect. All the cases number for different group extracted from each included literature were input to meta-analysis 3.13 software to calculate the pooled negative conversion rate (NCR), cure rate (CR) or vertical transmission rate based on their sample size and weight. The pooled NCR with 95% confidence intervals (CI) was used to evaluate the overall rate of a diagnosis positive result conversion to a negative result after treatment, which of spiramycin, azithromycin and TCM were 83.4% (95%CI, 72.1%-90.8%), 82.5% (95%CI, 75.9%-87.6%), and 85.5% (95%CI, 71.3%-93.3%) respectively, with no statistical difference between them. The pooled CR with 95% CI was used to evaluate the overall rate of complete disappearance of clinical symptoms for toxoplasmic encephalitis after therapy, which of P-S, TMP-SMX, and P-C were 49.8% (95%CI, 38. 8% -60.8%), 59.9% (95%CI, 48.9%-70.0%), and 47.6% (95%CI, 24.8%-71.4%) respectively, with no statistical difference between them. Primary *T*. *gondii* infection in pregnancy was treated mainly with spiramycin alone or combined with other drugs, and the pooled rate of vertical transmission was about 9.9% (95%CI, 5.9%-16.2%) after therapy. Toxoplasmic encephalitis in AIDS patients was usually treated with sulfonamides combined with other drugs and the pooled CR was 49.4% (95%CI, 37.9%-60.9%).

## Introduction

It is estimated that 1/3 of the world's population is infected with *Toxoplasma gondii* [1]. The infection is asymptomatic in most immunocompetent people, but serious consequences and even death may occur in immunocompromised patients. Foetal death, malformation or abortion may occur in pregnant women who have no previous exposure or immunity to *T*. *gondii* [2, 3]. Agents used to treat toxoplasmosis act against the replicating tachyzoite stage, which is metabolically active as it destroys tissues and disseminates throughout the patients’ bodies. Once a chronic infection of *T*. *gondii* becomes established, the tachyzoites produce latent tissue cyst stages. The tissue cyst stages contain bradyzoites, which are only minimally metabolically active. Most drugs used to treat *T*. *gondii* do not significantly affect the tissue cyst stage or the bradyzoites. The tissue cysts produced in the brain are protected by the blood-brain barrier, which makes treatment even more difficult. Furthermore, if the patients become immune suppressed, the tissue cysts may rupture, and the bradyzoites will transform back to rapidly multiplying tachyzoites, leading to a focal or diffuse encephalitis [4, 5].The most common used therapeutic medicines for *T*. *gondii* infection are pyrimethamine and sulfadiazine. For patients with hypersensitivity to sulfonamides, pyrimethamine alone in high dosages or in combination with clindamycin, clarithromycin, azithromycin or atovaquone can be used as substitutes [6, 7]. Other medicines, including spiramycin, azithromycin, trimethoprim plus sulfamethoxazole (TMP-SMX), are also commonly used. Traditional Chinese medicine (TCM) formulae containing herbs *Astragalus membranaceus*, *Scutellaria baicalensis*, *Artemisia annua*, *Ginkgo biloba etc* are also used in the treatment of *T*. *gondii* infections [8–10]. Spiramycin has minimal foetal toxicity and can be effectively absorbed after oral administration, spreading quickly to tissues to kill the tachyzoites and reduce the parasite or prevent the parasite from spreading through the placenta to the foetus [11]. Several studies revealed that maternal treatment with spiramycin alone or in combination with cotrimoxazole reduced the possibility of *T*. *gondii* vertical transmission and the disease sequelae in newborns, and delayed fetal infection [12–14], while it was also mentioned that the evidence was weak for early treatment in maternal infection reducing the risk of vertical transmission [15]. In the treatment of toxoplasmic encephalitis, pyrimethamine- sulfadiazine (P-S), which is often the first line of treatment, results in clinical or radiographic improvement in 70% to 90% of patients [7, 16]. Reports on the treatment of toxoplasmic encephalitis in AIDS patients also indicate that the efficacy of P-S, and pyrimethamine plus clindamycin (P-C) are not significantly different in the acute phase treatment, but in the maintenance phase of that treatment, relapse rate was twice as high among patients in the P-C treatment group [17]. A systematic review and meta-analysis on the treatment and control of toxoplasmic retinochoroiditis has been published, and there is a lack of evidence to support routine antibiotic treatment being effective for acute *Toxoplasma* retinochoroiditis [18, 19].

To resolve the uncertainty regarding the treatment efficacy of these drugs, we conducted a systematic review of the literature and a meta-analysis to explore the therapeutic effects of different drugs used in the treatment of *T*. *gondii* infection. Because different groups of people may need different therapeutic drugs, the treatments of *T*. *gondii* infection in pregnant women and toxoplasmic encephalitis patients were specifically analyzed here.

## Methods

### 2.1 Search strategy

Our study was performed according to the recommendations of the PRISMA Statement [20] (see S1 Checklist). PubMed, Google Scholar, Cochrane Library, Medline, Wanfang database, CNKI, and Chinese medical Citation Database, were searched combining the terms "*Toxoplasma gondii* infection" or "toxoplasmosis" and "treatment" or "therapy" or "cure" or "drug therapy".

### 2.2 Inclusion and exclusion criteria

Studies were included if they meet the criteria as follows: (i) it was a cohort study; (ii) clinical trials were based on humans, rather than animals; (iii) when more than one article was based on a study of the same population, the one with the highest quality (the Science Citation Index (SCI) was highest or the citations of the article were highest) was chosen; (iv) the diagnosis of *T*. *gondii* infection was based on a serological examination for *T*. *gondii* antibodies (IgA, IgG and /or IgM), parasite observation, or PCR detection of DNA; a positive result was represented by the presence of IgM, IgA, significant increase in specific IgG or a positive parasite observation, and a positive PCR result in detection of *T*. *gondii* DNA; a negative result was defined as a lack of IgM, IgA, a significantly dropped or absent titer of IgG, a negative PCR result when detecting *T*. *gondii* DNA, or a negative parasite observation. (v) for toxoplasmosis cure rate evaluation, only toxoplasmic encephalitis was included, *T*. *gondii* infection diagnosis refer to (iv), encephalitis was confirmed by computed tomography or magnetic resonance imaging scans, and clinical signs or symptoms included fever, headache, seizures, lethargy-coma, hemiplegia or hemiparesis and so on; (vi) the diagnosis of congenital *T*. *gondii* infection met one of the following standards: A. increase of specific IgG in the first 12 months of age or persistence of specific IgG antibodies beyond 7 months till 12 months of age; B. positive with *T*. *gondii* specific IgM and/or IgA in cord blood and/or in neonatal blood; C. presence of parasite in amniotic fluid, placenta or fetal blood confirmed by inoculation to mice ascites, or by PCR test. The vertical transmission rate represents the risk of congenital infection when a mother was infected by *T*. *gondii*.

All the cases number for different group extracted from each included literature were input to meta-analysis 3.13 software to calculate the pooled negative conversion rate (NCR) or cure rate (CR) or vertical transmission rate based on their sample size and weight. NCR is valuable in the assessment of the treatment effects in asymptomatic infections, and CR is intuitionistic in the evaluation of symptomatic infections. The pooled NCR shows the overall rate of a positive diagnosis conversion to a negative diagnosis of *T*. *gondii* antibody or DNA, with a treatment time of generally less than a month. The pooled CR of toxoplasmic encephalitis is defined as "the overall rate of complete absence of clinical symptoms of encephalitis after therapy".

Citations were excluded for the following reasons:(i) the paper was a review or a descriptive study; (ii) the data were duplicated from other studies or we were unable to obtain.

### 2.3 Data extraction

The following information was extracted from each study: first author, publication time, the sample size, diagnostic methods, drug and drug dosage. The data were collected independently by two investigators (H-X. W & S-S. W). When the literature citations were controversial, these investigators discussed them and reached a consensus on inclusion or exclusion.

### 2.4 Data analysis

As spiramycin, azithromycin and traditional Chinese medicine (TCM) were commonly used in the treatment of general population or pregnant women infection with *T*. *gondii* which were usually asymptomatic, their therapeutic efficacies were evaluated with negative conversion rate (NCR) after treatment. As pyrimethamine-sulfadiazine (P-S), trimethoprim-sulfamethoxazole (TMP-SMX), and pyrimethamine-clindamycin (P-C) were commonly used in the treatment of toxoplasmic encephalitis, their therapeutic efficacies as well as the effects of the treatments in AIDS patients with toxoplasmic encephalitis were evaluated with cure rate (CR). The risk of *T*. *gondii* vertical transmission after the treatment of primary infection in pregnant women was evaluated with the overall vertical transmission rate after maternal treatment. In this meta-analysis, the heterogeneity among the studies was tested by a χ^2^-based Q test or I^2^ statistic [21]. When p<0.05 or I^2^>50%, the heterogeneity was considered significant. A random-effects model was chosen to calculate the pooled NCRs or CRs or vertical transmission rate when the heterogeneity was significant, otherwise a fixed-effects model was used. Meta-analysis 3.13 software (Tufts Medical Centre, Boston, MA, USA) was used in this research. The therapeutic effects were compared with SPSS 13.0 software (Chicago, USA) between any two different drugs. The variance homogeneity of the extracted data was measured first. Analysis of variance (ANOVA) was conducted if it met the homogeneity of variance; otherwise, the rank sum test was used.

## Results

### 3.1 Analysis of the included literature

**Fig 1:**
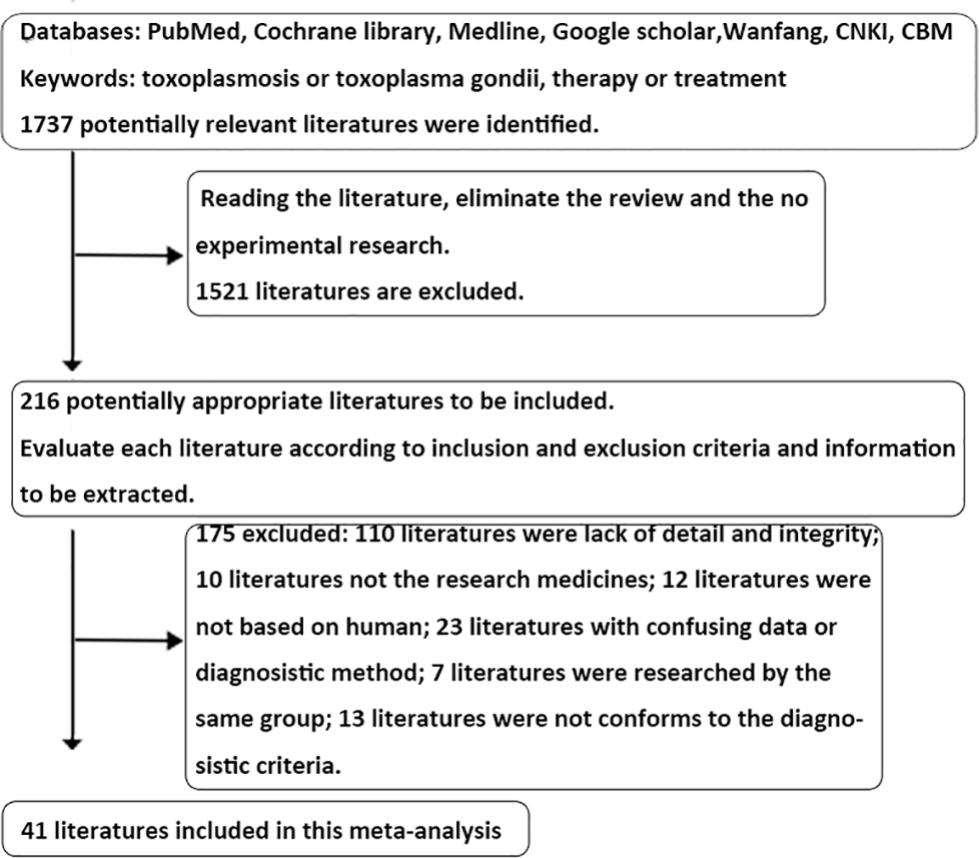
Flow diagram of the selection of the studies.

Forty-one publications matched the inclusion criteria, including 9 studies about the treatment with spiramycin [1–9 in S1 Appendix], 4 with azithromycin [8, 10–12 in S1 Appendix], 7 with TCM [5, 12–17 in S1 Appendix], 7 with pyrimethamine-sulfadiazine (P-S) [18–24 in S1 Appendix], 3 with trimethoprim-sulfamethoxazole (TMP-SMX) [18, 24, 25 in S1 Appendix], 6 with pyrimethamine-clindamycin (P-C) [19, 20, 26–29 in S1 Appendix], 11 concerning the risk of vertical transmission from the pregnant women in primary infection of *T*. *gondii* after treatment [6, 30–39 in S1 Appendix] and 14 about the treatment of toxoplasmic encephalitis in AIDS patients [18–29, 38, 39 in S1 Appendix]. Details about the first author, publication time, sample size, diagnostic methods and drug dosage are shown in Tables 1–8 in S1 Appendix. A flow diagram of the selection and nature of the studies is shown in Fig 1.

### 3.2 Quantitative synthesis and heterogeneity analysis

#### 3.2.1 The pooled negative conversion rate (NCR) of spiramycin, azithromycin and TCM treatment for *Toxoplasma gondii* infection

**Fig 2:**
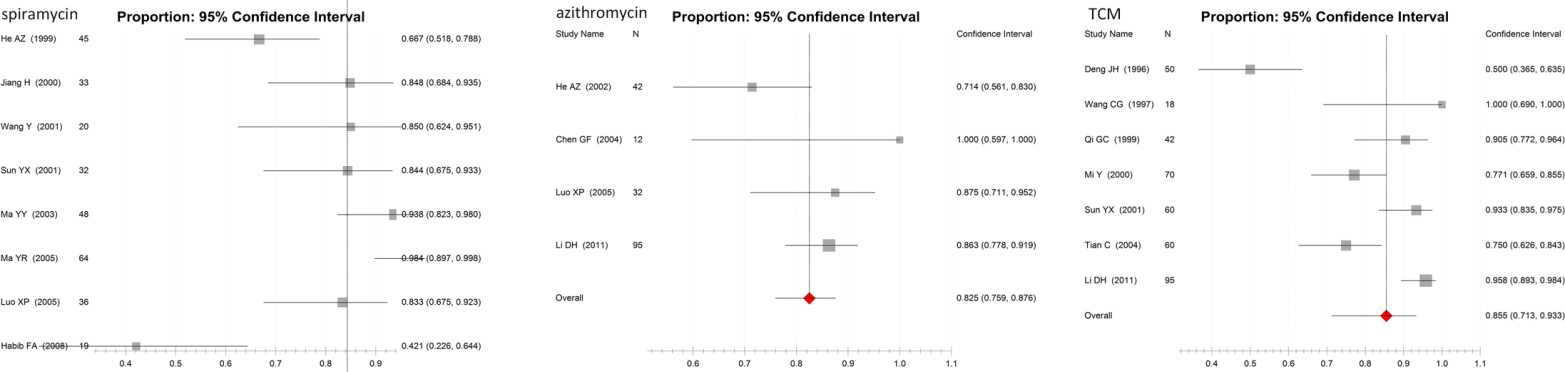
Forest plots of the therapeutic effects of different anti-*Toxoplasma gondii* medicines (spiramycin, azithromycin, TCM). Scale:NCR of drugs.

For spiramycin treatment, 9 publications with 315 participants were included (Table A in S1 Appendix); the NCR (95%CI) was 84.3% (71.9%-91.8%), calculated with the random-effects model (p = 0.000, I^2^ = 43.4%). For azithromycin treatment, 4 publications with 181 participants were included (Table B in S1 Appendix); the NCR (95%CI) was 82.5% (75.9%-87.6%), calculated with the fixed-effects model (p = 0.090, I^2^ = 34.3%). For TCM, 7 publications with 395 participants were included (Table C in S1 Appendix); the NCR (95%CI) was 85.5% (71.3%-93.3%), calculated with the random-effects model (p = 0.000, I^2^ = 46.6%). No statistically significant difference was found in the therapeutic effects of spiramycin, azithromycin, or TCM (p = 0.915 in the one-way ANOVA test for 3 independent-samples; p = 0.658 in the test of homogeneity of variances). All the details about the therapeutic effects in NCR of these medicines were shown in Fig 2.

#### 3.2.2 The pooled cure rate (CR) of P-S, TMP-SMX and P-C in toxoplasmic encephalitis treatment

**Fig 3:**
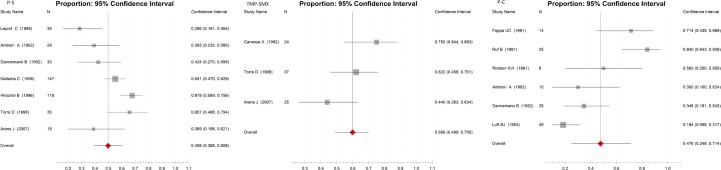
Forest plots of the therapeutic effects of different anti-Toxoplasma gondii medicines (P-S, TMP-SMX, and P-C). Scale: NCR of drugs.

For P-S treatment, 7 publications with 414 participants were included (Table D in S1 Appendix); the CR was 49.8% (95%CI, 38.8%-60.8%), calculated with the random-effects model (p = 0.000, I^2^ = 43.1%). For TMP-SMX treatment, 3 publications with 86 participants were included (Table E in S1 Appendix); the CR was 59.9% (95%CI, 48.9%-70.0%) calculated with the fixed-effects model (p = 0.083, I^2^ = 36.9%). For P-C treatment, 6 publications with 132 participants were included (Table F in S1 Appendix); the CR was 47.6% (95%CI, 24.8%-71.4%), calculated with the random-effects model (p = 0.000, I^2^ = 45.3%). No statistically significant difference was found in the CRs of P-S, P-C or TMP-SMX (p = 0.635 in the one-way ANOVA test for 3 independent samples; P = 0.238 in the test of homogeneity of variances). All the details about the therapeutic effects in CRs of these medicines were shown in Fig 3.

#### 3.2.3 The risk of vertical transmission after treatment for the pregnant women’s primary infection of *Toxoplasma gondii* and the therapeutic efficacies of different drugs for toxoplasmic encephalitis

**Fig 4:**
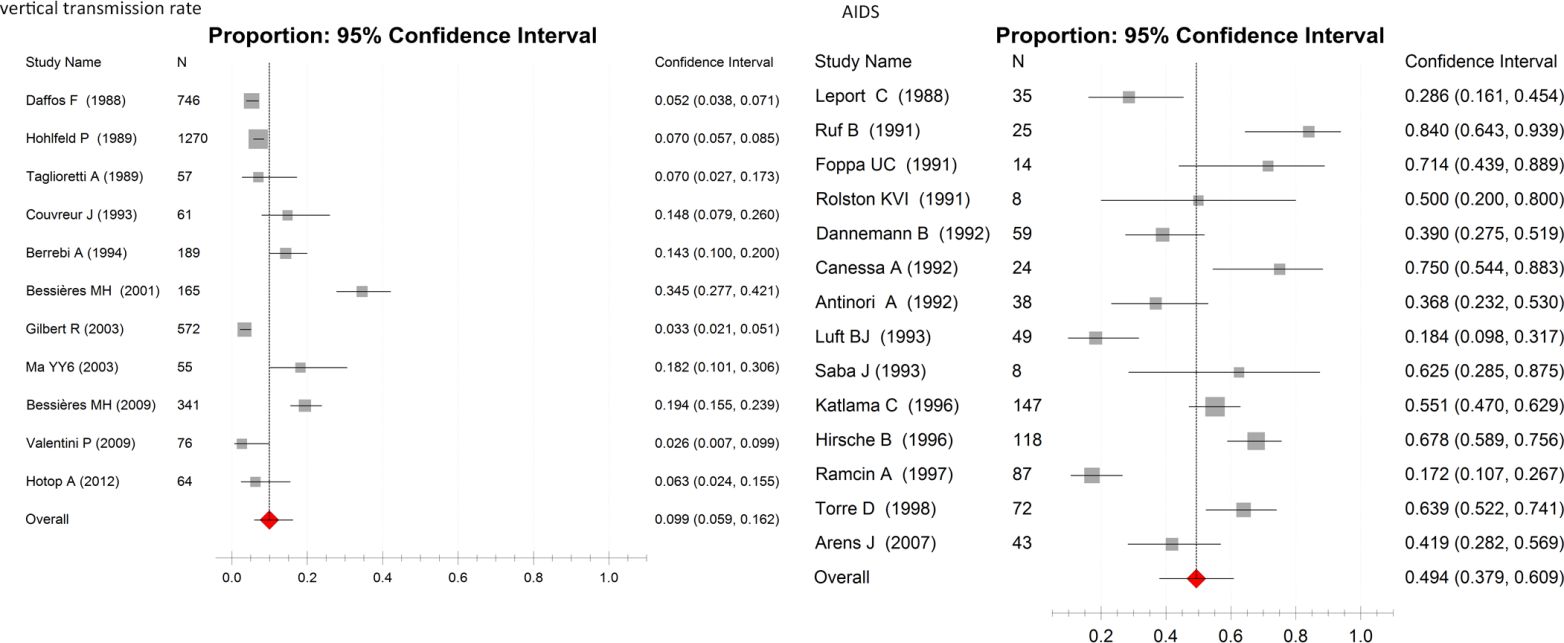
Forest plots of the therapeutic effects for vertical transmission rate and AIDS patients with *Toxoplasma* encephalitis (AIDS). Scale: NCR of drugs.

For the vertical transmission rate after treatment in the primary infection of pregnant women regardless of gestation age, 11 publications with 3596 participants were included treated with spiramycin alone or combined with P-S (Table G in S1 Appendix); the vertical transmission rate was 9.9% (95%CI, 5.9%-16.2%) with this therapy, which was calculated with the random-effects model (p = 0.000, I^2^ = 48.6%). This result showed that the chance of vertical transmission was approximately 9.9% when the infected mother received necessary treatment against *T*. *gondii* with spiramycin alone or combined with P-S. For the treatment of toxoplasmic encephalitis, 14 publications (Table H in S1 Appendix) with 727 participants were included. The pooled cure rate (CR) of these treatments was 49.4% (95%CI, 37.9%-60.9%), which was calculated with the random-effects model (p = 0.000, I^2^ = 46.5%). This result showed that approximately 49.4% of toxoplasmic encephalitis would be cured with different therapeutic regimes (Fig 4).

## Discussion

### 4.1 The efficacy of the therapeutic medicines in the treatment of T. gondii infection

#### 4.1.1 Spiramycin, azithromycin, and traditional Chinese medicine (TCM)

Spiramycin can be absorbed efficiently after oral administration and spreads to blood and tissues quickly, and there has been no evidence that spiramycin is teratogenic, so it can be used in the treatment of *T*. *gondii* infection in the general population and women in the first trimester of pregnancy to prevent the vertical transmission [22]. In the 9 included literatures, 5 of which were about the treatment of pregnant women infection, 4 of which were about the treatment of general population including male and female or women at childbearing age (Table A in S1 Appendix). Negative conversion rate (NCR) was used to evaluate the therapeutic efficacy. It was shown that the overall NCR of spiramycin treatment was 84.3% (95%CI: 71.9%-91.8%) in these groups of population.

Azithromycin was used less than spiramycin because of its potential liver toxicity [23]. In the 4 included literatures, 1 of them was about the therapy of the women at childbearing age, 2 of them were about the therapy of infant and child under 14 years old, 1 of male and female aged from 3–58 years (Table B in S1 Appendix). The pooled NCR was used to evaluate the therapeutic efficacy of azithromycin, which was 82.5% (95%CI, 75.9%-87.6%) in our study. As a new generation of macrolide, azithromycin can reduce the sequelae rate among the infected infants at age 1 year [24], and was widely used in the treatment of refractory ocular toxoplasmosis [25].

*Artemisia annua* was shown to reduce the number or density of *T*. *gondii* tachyzoites in host cells *in vitro* and *i*n vivo [8, 26, 27]. Other herbs, including *Phellodendron chinense*, *Hedyotis diffusa*, rhizoma *Anemarrhenae asphodeloides*, radix *Sophorae flavescentis*, fructus *Amomum tsaoko*, radix *Astragalus membranaceus*, *Semen arecae*, cortex *Moutan radicis Poria cocos*, *Glycyrrhiza uralensis*, *Rheum palmatum* were reported to be able to kill *T*. *gondii* tachyzoites and tissue cyst bradyzoites in different combination in host cells and in mice [28–34]. These herbs are the basic ingredients in formulations for anti-*T*. *gondii* TCMs. Along with these active ingredients, other personalised conditioning herbs are usually included in TCM treatments to enhance the immune response of the patient. In this study to evaluate the treatment efficacy of TCM against *T*. *gondii* infection, 7 literatures were included (Table C in S1 Appendix, Fig 2); the population involved children under 14 years old, men, women, and pregnant women. The pooled NCR of these TCMs was 85.5% (95%CI, 71.3%-93.3%). It was reported that a combination of antibiotics and TCMs for treatment of *T*. *gondii* infections showed a 100% NCR in 359 cases after 3 periods of treatment for 30 days [35, 36]. Controlled studies are needed to determine if well-defined formulations of the herbs used in TCM have activity against *T*. *gondii*, and it is also required to chemically define the active ingredients.

In the treatment for *T*. *gondii* infection in immune competent people which usually are asymptomatic, negative conversion rate is possible for the evaluation of the treatment efficacy. From the included literatures, we found that spiramycin, azithromycin and TCM were mostly used in the treatment of the infection in immune competent population. When they were used alone in treatment of *T*. *gondii* infection, no statistical difference was observed in the therapeutic efficacy of these medicines.

#### 4.1.2 pyrimethamine-sulfadiazine (P-S), trimethoprim-sulfamethoxazole (TMP-SMX) and pyrimethamine-clindamycin (P-C)

Cure rate (CR) was used to evaluate the therapeutic efficacy for the treatment of toxoplasmosis. In our study, all the included literatures for P-S, TMP-SMX, and P-C treatment were about toxoplasmic encephalitis in AIDS patients except one in P-C treatment, which was about the acute toxoplasmic encephalitis (Table D, E, F in S1 Appendix). The pooled CRs for P-S treatment was 49.8% (95%CI, 38. 8% -60.8%), for TMP-SMX treatment was 59.9% (95%CI, 48.9%-70.0%), and 64.7% (95%CI, 51.7%-75.8%) for P-C treatment, and no statistically significant difference was found in the CRs of P-S, P-C or TMP-SMX in toxoplasmic encephalitis therapy. It had been reported in a clinical treatment comparison that there was no statistically significant difference in efficacy during acute therapy between P-S and P-C, while P-C treatment got a relapse rate twice as high among patients comparing to P-S treatment [17]. A systematic review for assessment of the efficacy of different treatment regimens in toxoplasmic encephalitis with the randomized clinical trials showed that efficacy of P-S and TMP-SMX were similar, whereas P-S versus P-C showed no difference [37], which was consistent with our analysis. TMP-SMX suppresses the proliferation of the occasional parasites that emerge from tissue cysts [38]. Pyrimethamine acts on the folatebiosynthesis pathway, inhibiting dihydrofolate reductase activity and thus eventually blocking nucleic acid synthesis [39]. Pyrimethamine in combination with sulfadiazine is effective at inhibiting intracellular *T*. *gondii* [40]. From our study, the pooled cure rates of TMP-SMX and P-C were relatively higher than that of P-S in the treatment of toxoplasmic encephalitis. However, it was recommended that P-S is the best choice of treatment, because treatment with TMP-SMX and P-C had a higher recurrence rate, and P-C has more side-effects than P-S [17, 41, 42].

### 4.2 The efficacy of the different drugs in the treatment of *T*. *gondii* infection in different groups of people

#### 4.2.1 The risk of vertical transmission after treatment for the pregnant women’s primary infection of *Toxoplasma gondii*

Some studies showed that the risk of congenital toxoplasmosis is reduced in different degree when prenatal treatment was started early in pregnancy [15, 43, 44]. On the contrary, the SYROCOT found no evidence that prenatal treatment significantly reduced the risk of clinical manifestations in infected live born infants [15]. Despite this contradiction, most of the related studies showed that increasing gestational age was strongly associated with increased risk of mother-to-child transmission whatever treated or not [15,45].

Spiramycin had been recommended in the treatment for suspected or confirmed acute *T*. *gondii* infection in women during the first 18 weeks of gestation, and could reduce the vertical transmission rate [22, 46, 47], but the treatment medicine should be switched to pyrimethamine-sulfadiazine (P-S) added with folinic acid after 18 weeks of gestation [48]. One reason is that the risk of fetal infection increased with the gestation age, as the placental barrier in later trimesters is less efficient than at the beginning of pregnancy [49]. The other reason is because spiramycin cannot pass through the placenta to the foetus, the treatment of intrauterine infections is not efficient [50, 51]. Generally speaking, rapid initiation of therapy to the infected pregnant women is still important, because fetus infection follows shortly after the mother infection [15]. If foetal infection was confirmed by amniocentesis diagnosis, or if the mother was infected in late pregnancy and there was a high risk of intrauterine infection, P-S with folic acid or its derivatives should be used to the reduce the risk of congenital toxoplasmosis [50, 51].

In our study, to know about the risk of vertical transmission after therapy, the included literatures were 3 papers about spiramycin alone, 5 about the combination of spiramycin and P-S, and 3 about the combination of spiramycin and P-S and folinic acid. It was shown that the mother-to-child transmission rate was 9.9% (95%CI, 5.9%-16.2%) after treatment in primary infection regardless of gestation age. Romand S reported that the overall rate of congenital transmission in maternal infection of *T*. *gondii* regardless of gestational age was about 28%, and 97% of the maternal infection had been treated with spiramycin[52]. The difference of the vertical transmission rates between our study and Romand S’ study may due to the different drugs used in treatment and the included samples. All of the samples in our study were treated with spiramycin alone or combined with P-S, while in Romand S’ study, the samples were treated or not with spiramycin. Because the papers included in our study are about patients who received treatment, we don’t know the vertical transmission rate of patients who did not receive any treatment. Randomized and control studies are needed to determine the efficacy of treatment in reducing the mother-to-child transmission rate.

#### 4.2.2 The therapeutic efficacies of different drugs for toxoplasmic encephalitis in AIDS patients

In the 14 included papers for the treatment of toxoplasmic encephalitis in AIDS patients, pyrimethamine-sulfadiazine (P-S) was used in 8 papers, pyrimethamine-clindamycin (P-C) was adopted in 5 papers, trimethoprim-sulfamethoxazole (TMP-SMX) was used in 3 papers, clindamycin alone, pyrimethamine-azithromycin and atovaquone alone were used in 1 paper respectively. The overall cure rate (CR) of toxoplasmic encephalitis in AIDS patients was only 49.4% with different therapeutic regimes, which may due to the inability of the anti-*T*.*gondii* medicines to cross the blood-brain barrier and reach the central nervous system. P-S and TMP-SMX had apparent effects, though limited, on primary prevention (chemoprophylaxis to prevent first episodes of opportunistic infections) of toxoplasmosis in AIDS patients with mild allergic reactions and side-effects [53]. Very few drugs were efficient in secondary prevention (chronic maintenance therapy) of toxoplasmic encephalitis, and P-S was commonly used in this situation [54]. Azithromycin alone or combined with pyrimethamine or SMZ-TMP was also a relatively safe and effective choice for secondary prevention when P-S was unavailable or contraindicated [55].

## Conclusions

The efficacies of spiramycin, azithromycin, traditional Chinese medicine (TCM), pyrimethamine-sulfadiazine (P-S), trimethoprim-sulfamethoxazole (TMP-SMX), and pyrimethamine-clindamycin combination (P-C) to treat *T*. *gondii* infections were examined in this meta-analysis. Generally speaking, spiramycin, azithromycin and TCM were commonly used in the therapy of general populations infected with *T*. *gondii*, had a negative conversion rate of 83.4% (95%CI, 72.1%-90.8%), 82.5% (95%CI, 75.9%-87.6%), and 85.5% (95%CI, 71.3%-93.3%), respectively, and no statistical difference was found between these medicines in therapeutic efficacy. P-S, TMP-SMX and P-C were usually used in the therapy of toxoplasmic encephalitis with the cure rates (CR) of 49.8% (95%CI, 38. 8% -60.8%), 59.9% (95%CI, 48.9%-70.0%), and 47.6% (95%CI, 24.8%-71.4%), respectively, and these were also not significantly different in statistics. Primary infection of *T*. *gondii* in pregnant women were treated mainly with spiramycin alone or combined with P-S and/or folic acid, and the vertical transmission was 9.9% (95%CI, 5.9%-16.2%) after therapy. Sulfonamides combined with other drugs were mostly used in the treatment of toxoplasmic encephalitis in AIDS patients; however, the pooled CR was only 49.4% (95%CI, 37.9%-60.9%) in different therapeutic regimes.

## Supporting Information

S1 Checklist PRISMA 2009 checklist for the manuscript. (DOC)

S1 Appendix Details of each analysis and references of 41 included literatures. Characteristics of the individual studies included in spiramycin treatment **(Table A)**. Characteristics of the individual studies included in azithromycin treatment **(Table B)**. Characteristics of the individual studies included in traditional Chinese medicine treatment group **(Table C)**. Characteristics of the individual studies included in pyrimethamine- sulfadiazine (P-S) treatment **(Table D).** Characteristics of the individual studies included in trimethoprim-sulfamethoxazole (TMP-SMX) treatment **(Table E)**. Characteristics of the individual studies included in pyrimethamine-clindamycin (P-C) treatment **(Table F)**. Details of included studies about the risk of vertical transmission after the treatment of primary Toxoplasma gondii infection in pregnant women **(Table G)**. Characteristics of the individual studies included in the treatment of toxoplasmic encephalitis in AIDS patients **(Table H).**
(DOC)

## Abbreviations

P-S: pyrimethamine and sulfadiazine combination
P-C: pyrimethamine and clindamycin combination
TMP-SMX: trimethoprim and sulfamethoxazole combination
TCM: traditional Chinese medicine
NCR: negative conversion rates
CI: confidence interval
CR: cure rate
