# Supplementary material for: A Systematic Review and Meta-Analysis of the Efficacy of Anti-Toxoplasma gondii Medicines in Humans
Source: PLoS One. 2015 Sep 22;10(9):e0138204. doi: 10.1371/journal.pone.0138204 (PMC4578932; doi:10.1371/journal.pone.0138204)
Supplement: S1 Appendix — Characteristics of the individual studies included in spiramycin treatment (Table A). Characteristics of the individual studies included in azithromycin treatment (Table B). Characteristics of the individual studies included in traditional Chinese medicine treatment group (Table C). Characteristics of the individual studies included in pyrimethamine- sulfadiazine (P-S) treatment (Table D). Characteristics of the individual studies included in trimethoprim-sulfamethoxazole (TMP-SMX) treatment (Table E). Characteristics of the individual studies included in pyrimethamine-clindamycin (P-C) treatment (Table F). Details of included studies about the risk of vertical transmission after the treatment of primary Toxoplasma gondii infection in pregnant women (Table G). Characteristics of the individual studies included in the treatment of toxoplasmic encephalitis in AIDS patients (Table H). (DOC) [file pone.0138204.s002.doc]

**S1_Appendix**：**Details of each analysis and references of 41 included literatures.**

**Table A Characteristics of the individual studies included in s**piramycin treatment

| First author | year | diagnostic methods | People | Negative cases number | Positive cases number | Drug dosage |
| --- | --- | --- | --- | --- | --- | --- |
| Bao YL^1^ | 1994 | IHA-Serological antibody( IgG)* | 27 male and 33 female, aged from 14 to 67 years, from October 1989 to March 1992. | 18 | 0 | 1 .6 ~ 2g /d |
| He AZ^2^ | 1999 | TOX-IgM, DNA | 24 male and 21 female, aged from 1 to 14 years, from January 1996 to December 1998. | 30 | 15 | 40m/kg.d |
| Jiang H^3^ | 2000 | TOX-IgM, DNA | Pregnant women, aged from 22 to 36 years, from March 1996 to March 1998. | 28 | 5 | 0.5 g／6 h, for 10d |
| Wang Y^4^ | 2001 | TOX-IgM | Pregnant women, aged from 22 to 34 years, from March 1998 to March 2000. | 17 | 3 | 0.5 g / 6 h, for 10d |
| Sun YX^5^ | 2001 | Tox-DNA | Women at childbearing age, aged from 23 to 29 years, from January 2000 to December 2000. | 27 | 5 | 1.2g/d，for 21d |
| Ma YY^6^ | 2003 | Tox-DNA | Pregnant women, aged from 23 to 41 years, from January 1999 to December 2001. | 45 | 3 | 3g/d, for 20d |
| Ma YR^7^ | 2005 | TOX-IgM | Pregnant women, January 1999 to December 2003. | 63 | 1 | 2g/d, for 14d |
| Luo XP^8^ | 2005 | TOX-IgM | Women at childbearing age, aged from 23 to 38 years, from January 2001 to October 2004. | 30 | 6 | 2g/d, for 14d |
| Habib FA^9^ | 2008 | Tox-DNA | Pregnant women | 8 | 11 | unknown |

*: The antibody titer >1：64

**Table B Characteristics of the individual studies included in azithromycin treatme**nt

| First author | year | diagnostic methods | People | Negative cases  number | Positive cases number | Drug dosage |
| --- | --- | --- | --- | --- | --- | --- |
| He AZ^10^ | 2002 | TOX-IgM, Tox-DNA | 42 male and 38 female, aged from 1 to 14 years. | 30 | 12 | 7-10mg/kg.d, for 30d |
| Chen GF^11^ | 2004 | TOX- IgM, Tox-DNA | Male and female, aged from 1 month to 7 months, from 1997 to 2003. | 12 | 0 | 10mg/kg.d, for 25d |
| Luo XP^8^ | 2005 | TOX-IgM | Women at childbearing age, aged from 23 to 38 years, from January 2001 to October 2004. | 28 | 4 | 0.5g/d, for 14d |
| Li DH^12^ | 2011 | parasite observation | patients with toxoplasmosis, male and female aged from 3-58 years. | 82 | 13 | 0.75g/d, for 45d |

**Table C Characteristics of the individual studies included in traditional Chinese medicine treatment group**

| First author | year | diagnostic methods | People | Negative cases  number | Positive cases number | Ingredients |
| --- | --- | --- | --- | --- | --- | --- |
| Deng JH^13^ | 1996 | TOX-IgG or IgM | 33 males and 17 females, aged from 1-14 years old, 1984-1995. | 25 | 25 | radix *Astragalus membranaceus*, *Artemisia annua*, fructus *Amomum tsaoko*, *Semen arecae*, rhizoma *Atractylodes macrocephala* |
| Wang CG^14^ | 1997 | TOX-IgM | 18 people (women or men) who were recently infected. | 18 | 0 | *Artemisia annua* |
| Qi GC^15^ | 1999 | Tox-DNA | Men infertility, aged from 24-41 years old | 38 | 4 | *Sargent gloryvine*, radix *Salviae miltiorrhizae*, *Hedyotis diffusa*, semen *Vaccaria segetalis*, semen *Plantago asiatica*, *Phellodendron Chinense*, rhizoma *Anemarrhenae asphodeloides*, radix *Rheum palmatum*, cortex *Moutan radicis* |
| Mi Y^16^ | 2000 | TOX-IgM | Pregnant women, aged from 24-39 years old, 1992-1998. | 54 | 16 | radix *Astragalus membranaceus*, radix *Panax ginseng*, radix *Engelicae sinensis*, radix *Paeoniae alba*, *Artemisia annua*, fructus *Amomum tsaoko*, *Semen arecae*, *Phellodendron Chinense*, rhizoma *Smilacis glabrae*, *Hedyotis diffusa*, *Polygonum cuspidatum*, radix *Glycyrrhiza uralensis* |
| Sun YX^5^ | 2001 | Tox-DNA | Women at childbearing age, aged from 21-30 years old, Jan.2000- Dec.2000. | 56 | 4 | radix *Codonopsis purpurea*, radix *Astragalus membranaceus*, *Poria cocos*, rhizoma *Atractylodes macrocephala*, radix *Glycyrrhiza uralensis*, *Scutellaria baicalensis*, *Herba taraxaci*, *Lonicera japonica*, *Viola philipica* , Radix *Semiaquilegia adoroides*, radix *Sophorae flavescentis*, *Zanthoxylum bungeanum*, cortex *Moutan radicis* |
| Tian C^17^ | 2004 | Tox-DNA or  Tox-IgM | 36 men and 24 women, aged from 2-50 years old. | 45 | 15 | radix *Astragalus membranaceus*, *Artemisia annua*, *Poria cocos*, *Magnolia officinalis*, |
| Li DH^12^ | 2011 | parasite observation | patients with toxoplasmosis, male and female aged from 3-58 years. | 91 | 4 | *Antifebrile dichroa*, radix *Pulsatilla adans*, *Artemisia annua*, *Hairyvein agrimonia*, cortex *Periplocae radicis*, radix *Sophorae flavescentis*, *Flos chrysanthemi*, *Omphalia lapidescens*, *Pericarpium granat*, *Fructus cnidii*, *Fructus bruceae*, *Fructus mume*, radix *Rheum palmatum*, radix *Glycyrrhiza uralensis* |

**Table D Characteristics of the individual studies included in pyrimethamine****- sulfadiazine (P-S) treatment**

| First author | year | People | symptomatic | cured | sum | Drug dosage |
| --- | --- | --- | --- | --- | --- | --- |
| Leport C^18^ | 1988 | AIDS patients with toxoplasmic encephalitis | +(NA) | 10 | 35 | P: 100-200mg/d,1-2d,then 50-100mg/d,S:2-6g/d |
| Antinori A^19^ | 1992 | AIDS patients with toxoplasmic encephalitis | +(headache and/or fever (mild), focal neurological signs  (moderate), stupor and/or coma (severe)) | 11 | 28 | S:4-6g/d;P:50-75mg/d |
| Dannemann B^20^ | 1992 | AIDS patients with toxoplasmic encephalitis | +(NA) | 14 | 33 | S:100mg/kg.d,P: 200mg/d,then P 75mg/d. |
| Katlama C^21^ | 1996 | AIDS patients with toxoplasmic encephalitis | +(fever(102) and/or neurological symptoms or signs(headache(94); seizures(27); hemiplegia(53); aphasia(37); visual disorders(16) | 81 | 147 | P：50 mg /d, S: 4 g/d, for 6 weeks. |
| Hirschel B^22^ | 1996 | AIDS patients with toxoplasmic encephalitis, Jul. 1992- Dec.1994. | +(NA) | 80 | 118 | unknown |
| Torre D^23^ | 1998 | AIDS patients with toxoplasmic encephalitis without pregnant, age over 18 years old. | +(fever(21) and/or neurological symptoms or signs(headache(26); seizures(8); hemiplegia(17)) | 23 | 35 | P : 50 mg/d,S:60 mg/kg/d |
| Arens J^24^ | 2007 | AIDS patients with toxoplasmic encephalitis, 1993- 2003. | +(disturbance of consciousness(18); seizures(10) | 7 | 18 | P-S ,the dose unknown |

**Table E Characteristics of the individual studies included in** **trimethoprim-sulfamethoxazole (TMP-SMX) treatment**

| First author | year | People | symptomatic | cured | sum | Drug dosage |
| --- | --- | --- | --- | --- | --- | --- |
| Canessa A^25^ | 1992 | AIDS patients with toxoplasmic encephalitis | +( hemiparesis, lethargy, seizures, ataxia, coma) | 18 | 24 | 40 mg/kg/d (12 patients) or 120 mg/kg/d (12 patients) |
| Torre D^23^ | 1998 | AIDS patients with toxoplasmic encephalitis without pregnant, age over 18 years old. | +(fever(21) and/or neurological symptoms or signs(headache(21); seizures(14); hemiplegia(22)) | 23 | 37 | 6.6 to 20 mg/kg/d). |
| Arens J^24^ | 2007 | AIDS patients with toxoplasmic encephalitis, 1993- 2003. | +(disturbance of consciousness(26);seizures(17)) | 11 | 25 | unknown |

**Table F Characteristics of the individual studies included in** **pyrimethamine-clindamycin (P-C) treatment**

| First author | year | People | symptomatic | cured | sum | Drug dosage |
| --- | --- | --- | --- | --- | --- | --- |
| Foppa UC^26^ | 1991 | AIDS patients with toxoplasmic encephalitis | NA | 10 | 14 | CL:600-900mg,P100mg;then P 51mg/d |
| Ruf B^27^ | 1991 | AIDS patients with toxoplasmic encephalitis | NA | 21 | 25 | CL :1200-2400mg/d,Pri :50-75mg/d |
| Rolston KVI^28^ | 1991 | Patients with acute toxoplasmic encephalitis | NA | 4 | 8 | P :25mg/d,CL: 1200-2400mg/d |
| Antinori A^19^ | 1992 | AIDS patients with toxoplasmic encephalitis | +(headache and/or fever (mild), focal neurological signs (moderate), stupor and/or coma (severe)) | 3 | 10 | CL :2400-3600mg/d,P :50-75mg/d |
| Dannemann B^20^ | 1992 | AIDS patients with toxoplasmic encephalitis | +(NA) | 9 | 26 | CL:1200 mg/d, P:200 mg then 75 mg/d |
| Luft BJ^29^ | 1993 | AIDS patients with toxoplasmic encephalitis | +(fever; neurological symptoms or signs(headache; seizures; hemiplegia) | 9 | 49 | CL:600mg/6h,P: 200mg then 75mg/d |

**Table G Details of included studies about the risk of vertical transmission after the treatment of primary *Toxoplasma gondii* infection in pregnant women**

| First author | year | Trimester | Inclusion Criteria (mother) | Inclusion Criteria (baby) | regimen | No of infected children | No of not infected children |
| --- | --- | --- | --- | --- | --- | --- | --- |
| Daffos F^30^ | 1988 | all trimesters | seroconversion | IgM immunosorbent assay, a quantitative of maternal and fetal IgG, the inoculation of fetal blood into mice | spiramycin and P-S and folinic acid | 39 | 707 |
| Hohlfeld P^31^ | 1989 | all trimesters | seroconversion | IgM immunosorbent assay, a quantitative of maternal and fetal IgG, the inoculation of fetal blood into mice | Spiramycin alone | 89 | 1181 |
| Taglioretti A^32^ | 1989 | all trimesters | clinical and serological parameters | amniotic fluid and fetal blood detection of anti Toxoplasma IgM and isolation of the parasite. | Spiramycin alone | 4 | 53 |
| Couvreur J^33^ | 1993 | all trimesters | parasitological and serological signs | parasitological and serological signs | spiramycin and P-S | 9 | 52 |
| Berrebi A^34^ | 1994 | all trimesters | acute toxoplasma infection before 28 weeks of amenorrhoea | N/A | spiramycin and P-S | 27 | 162 |
| Bessières MH^35^ | 2001 | all trimesters | seroconversion | a positive parasitological examination by inoculation of mice with placenta or cord blood and / or detection of specific IgM and / or IgA antibodies in the cord blood with further confirmation by assaying blood samples taken on day 10 postpartum. | spiramycin and P-S | 57 | 108 |
| Gilbert R^36^ | 2003 | first trimesters | seroconversion | Congenital infection status confirmed by toxoplasma IgG results at one year postnatal age. | Spiramycin alone | 19 | 553 |
| Ma YY^6^ | 2003 | all trimesters | Tox-DNA | DNA positive | Spiramycin alone | 10 | 45 |
| Bessières MH^37^ | 2009 | all trimesters | Tox-DNA | Neonatal screening is based on the detection of parasites in the placenta and on the detection of IgM and IgA antibodies | Spiramycin and P-S | 66 | 275 |
| Valentini P^38^ | 2009 | <14 weeks | seroconversion | One of the following was needed: (1)seropositively for specific IgM and/or IgA in the first 6 months; (2)increase of specific IgG in the first 12 months of age; (3)persistence of specific IgG antibodies beyond 7 months of age, until 12 months; (4)clinical signs of infection | Spiramycin and P-S and folinic acid | 2 | 74 |
| Hotop A^39^ | 2012 | up to the beginning of the 16th week of pregancy | seroconversion | (1)increase of specific IgG in the first 12 months of age; (2)persistence of specific IgG antibodies beyond 7 months of age, until 12 months; (3)seropositively for specific IgM and/or IgA in the first 6 months; (4)detection of the pathogen by PCR in cord tissue and cord blood;(different IgG load compared to maternal IgG antibodies and additional IgG-reactive antibodies as shown in the comparative IgG immunoblot) | Spiramycin and P-S and folinic acid | 4 | 60 |

**Table H Characteristics of the individual studies included in the treatment of toxoplasmic encephalitis in AIDS patients**

| First author | year | People | symptoms | cured | sum | Drug dosage |
| --- | --- | --- | --- | --- | --- | --- |
| Leport C^18^ | 1988 | AIDS patients with toxoplasmic encephalitis | +(NA) | 10 | 35 | pyrimethamine: 100-200mg/d,1-2d,then 50-100mg/d, sulfadiazine :2-6g/d **(P-S)** |
| Ruf B^27^ | 1991 | AIDS patients with toxoplasmic encephalitis | NA | 21 | 25 | clindamycin:1200-2400mg/d; then 50-75mg/d |
| Foppa UC^26^ | 1991 | AIDS patients with toxoplasmic encephalitis | NA | 10 | 14 | pyrimethamine:100mg, clindamycin: 600-900mg, then pyrimethamine 51mg/d **(P-C)** |
| Rolston KVI^28^ | 1991 | Patients with acute toxoplasmic encephalitis | NA | 4 | 8 | pyrimethamine :25mg/d, clindamycin: 1200-2400mg/d **(P-C)** |
| Dannemann B^20^ | 1992 | AIDS patients with toxoplasmic encephalitis. | +(NA) | 23 | 59 | sulfadiazine:100mg/kg/d, pyrimethamine: 200mg/d, then pyrimethamine 75mg/d. or clindamycin:1200 mg/d, pyrimethamine:200 mg then 75 mg/d  **(P-C, P-S)** |
| Canessa A^25^ | 1992 | AIDS patients with toxoplasmic encephalitis | +( hemiparesis, lethargy, seizures, ataxia, coma,) | 18 | 24 | TMP-SMX: 40mg/kg/d (12  patients) or 120 mg/kg/d (12 patients) |
| Antinori A^19^ | 1992 | AIDS patients with toxoplasmic encephalitis | +(headache and/or fever (mild), focal neurological signs (moderate), stupor and/or coma (severe) | 14 | 38 | sulfadiazine:4-6g/d; pyrimethamine:50-75mg/d or clindamycin:2400-3600mg/d, pyrimethamine:50-75mg/d  **(P-C, P-S)** |
| Luft BJ^29^ | 1993 | AIDS patients with toxoplasmic encephalitis | +(fever; neurological symptoms or signs (headache; seizures ;hemiplegia) | 9 | 49 | clindamycin: 600mg/6h, pyrimethamine :200mg then 75mg/d  **(P-C)** |
| Saba J^40^ | 1993 | AIDS patients with toxoplasmic encephalitis | +(Fever, headache, ataxia, confusion, cerebella syndrome, obtundation, hemiparesia, paresia of the VI cranial nerve, pyramidal syndrome, seizure, dizziness) | 5 | 8 | 75 mg pyrimethamine and 500 mg azithromycin daily for four weeks (P-AZ) |
| Katlama C^21^ | 1996 | AIDS patients with toxoplasmic encephalitis | fever(102) and/or neurological symptoms or signs(headache(94); seizures(27); hemiplegia(53); aphasia(37); visual disorders(16)) | 81 | 147 | Pyrimethamine: 50 mg /d, sulfadiazine: 4 g/d, for 6 weeks **(P-S)** |
| Hirsche B^22^ | 1996 | AIDS patients with toxoplasmic encephalitis, July 1992 to December 1994. | +(NA) | 80 | 118 | Folinic acid associated with high doses of **P-S** |
| Ramcin A^41^ | 1997 | AIDS patients with toxoplasmic encephalitis, age over 13 years. | +(headache(45);seizure(14);impaired consciousness(23)) | 15 | 87 | atovaquone 3g/d |
| Torre D^18^ | 1998 | AIDS patients with toxoplasmic encephalitis without pregnant, age over 18 years. | +(fever(21) and/or neurological symptoms or signs(headache(26); seizures(8); pemiplegia(17)) | 46 | 72 | pyrimethamine: 50 mg/d, sulfadiazine:60 mg/kg/d **(P-S)** or TMP-SMX: 6.6 to 20 mg/kg/d |
| Arens J^24^ | 2007 | AIDS patients with toxoplasmic encephalitis, from 1993 to 2003. | +(disturbance of consciousness(18);seizures(10) | 18 | 43 | **P-S** or TMP-SMX, the dose unknown |

References:

1. Boa YL, Sai X, He Y, Yang CY, Lu S. (1994) The clinical analysis of 60 cases with obtained toxoplasmosis. Basic& Clinical Medicine 14：73-75.

2. He AZ, Yao J. (1999) The curative effects of azithromycin and interferon combination to treat 91 cases with toxoplasmosis. Chinese Journal of Zoonoses 16: 114-115.

3. Jiang H, Wen LZ, Ling XZ, Chen SH. (2000) The clinical evaluation of curative effect of acetylspiramycin on toxoplasmosis infection during pregnancy. Chinese Journal of Practical Gynecology and Obstetrics 16：107-108.

4. Wang Y, Li AQ. (2001) The treating of acetylspiramycin on 20 cases with toxoplasmosis in pregnancy. Shandong Medical Journal 41: 68-68.

5. Sun YX，Niu HZ, Yu LX. (2001) Righting Disinfection Treatment 60 cases toxoplasmosis of women in childbearing age. Chinese Journal of Integrated Traditional and Western Medicine 467-468.

6. Ma YY, Mu RL, Wang LY, Jiang S. (2003) Study on prenatal diagnosis using fluorescence quantitative polymerase chain reaction for congenital toxoplasmosis. Chinese Journal of Obstetrics and Gynecology 38:8-10.

7. Ma YR, Kong XY. (2005) The analysis of curative effects of acetylspiramycin on toxoplasmosis. Chinese journal of parasitic disease control 18: 216-216.

8. Luo XP, Luo XH, Wang AH. (2005) Compare the effects of two drugs treating women toxoplasmosis in childbearing age. Ch in J P arasi t Dis Con 18: 315-315.

9. Habib FA. (2008) Post-treatment assessment of acute Toxoplasma infection during pregnancy. J Obstet Gynaecol 28(6):593-5.

10. He AZ, Wang N. (2002) The treating of 80 cases with toxoplasmosis in respiratory with azithromycin and interferon combined. Chinese Journal of Zoonoses 18: 128-60 .

11. Chen GF, Fang YH, Guo DX, Feng XW, Xiang W, Ruan HQ. (2004) Follow up of 16 cases with congenital toxoplasmosis treated with azithromycin. Zhonghua Er Ke Za Zhi 42: 25-27.

12. Li DH, Li YG, Zhang WJ. (2011) Clinical studies on the treatment of toxoplasmosis by the anti- toxoplasmosis decoction. Journal of Pathogen Biology 6：295-296.

13. Deng JH, Fu L, Chen YS, Guo HF, Zhang JL. (1996) The soup of toxoplasmosis to treating 50 cases with toxoplasmosis in children. Journal of Traditional Chinese Medicine 37: 102-103.

14 Wang CG. (1997) The research of treating toxoplasmosis using *Artemisia annua* and preliminary observations clinical treatment. Chinese Journal of Zoonoses 13：79-80.

15. Qi GC, Li QX, Kan QL, Tong HW, Zhang GZ, Zhao JS, et al. (1999) The analysis of clinical curative effects of Qing Jing Granules on toxoplasmosis of 42 cases with infertility. Journal of Traditional Chinese Medicine 40：603-605.

16. Mi Y, Mi C. (2000) The treating of 70 cases toxoplasmosis in pregnancy using Astragalusannua. Shandong Journal of Traditional Chinese Medicine 19：83-84.

17. Tian C, Wang WH, Yuan XH, Ren LJ, Zhang XB, Zhang XM. (2004) The clinical study of righting eliminate the original pill on toxoplasmosis. Shandong Journal of Traditional Chinese Medicine 23: 528-529.

18. Leport C, Raffi F, Matheron S, Katlama C, Regnier B, Saimot AG, et al. (1988) Treatment of central nervous system toxoplasmosis with pyrimethamine/sulfadiazine combination in 35 patients with the acquired immunodeficiency syndrome. Efficacy of long-term continuous therapy. Am J Med 84:94-100.

19. Antinori A, Ammassari A, Maiuro G, Camilli G, Damiano F, Federico G, et al. (1992) Comparison of two medications in central nervous system toxoplasmosis in patients with AIDS. ltal J Neurol Sci 13:475-479.

20. Dannemann B, McCutchan JA, Israelski D, Antoniskis D, Leport C, Luft B, et al. (1992) Treatment of toxoplasmic encephalitis in patients with AIDS. A randomized trial comparing pyrimethamine plus clindamycin to pyrimethamine plus sulfadiazine. The California Collaborative Treatment Group. Ann of Intern Med 116:33–43.

21. Katlama C, De Wit S, O'Doherty E, Van Glabeke M, Clumeck N. (1996) Pyrimethamine-clindamycin vs. pyrimethamine-sulfadiazine as acute and longterm therapy for toxoplasmic encephalitis in patients with AIDS. Clinical Infectious Diseases 22: 268-275.

22. Bernard Hirschel, Christian Van Delden. (1996) Folinic Acid Supplements to Pyrimethamine-Sulfadiazine for Toxoplasma Encephalitis Are Associated with Better Outcome. The Journal of Infectious Diseases 173:1294-5.

23. Torre D, Casari S, Speranza F, Donisi A, Gregis G, Poggio A, et al. (1998) Randomised trial of trimethoprim sulfamethoxazole versus pyrimethamine-sulfadiazine for therapy of toxoplasmic encephalitis in patients with AIDS.Italian Collaborative Study Group. Antimicrobial Agents and Chemotherapy 42:1346–1349.

24. Arens J,  Barnes K, Crowley N, Maartens G. **(**2007) Treating AIDS-associated cerebral toxoplasmosis - pyrimethamine plus sulfadiazine compared with cotrimoxazole, and outcome with adjunctive glucocorticoids. S Afr Med J 97: 956-8.

25. Canessa A, Del Bono V, De Leo P, Piersantelli N, Terragna A. (1992) Cotrimoxazole therapy of Toxoplasma gondii encephalitis in AIDS patients. Eur J Clin Microbiol Infect Dis 11(2):125-30.

26. Foppa UC, Bini T, Gregis G, Lazzarin A, Esposito R, Moroni M. (1991) A retrospective study of primary and mainten ance therapy of toxoplasmic encephalitis with oral clindamycin and pyrimethamine. Eur J ClinMicrobiol Infect Dis 10:187-189.

27. Ruf B, Pohle HD. (1991) Role of clindamycin in the treatment of acute toxoplasmosis of the central nervous system. Eur J ClinMicrobiol Infect Dis 10:183-186.

28. Rolston KVI. (1991) Treatment of acute toxoplasmosis with oral clindamycin. Eur J ClinMicrobiol Infect Dis 10:181-183.

29. Luft B J, Hafner R, Korzun AH, Leport C, Antoniskis D, Bosler EM, et al. (1993) Toxoplasmic encephalitis in patients with the acquired immunodeficiency syndrome. Members of the ACTG 077p/ANRS 009 Study Team. NEJM 329: 995-1000.

30. Daffos F, Forestier F, Capella-Pavlovsky M, Thulliez P, Aufrant C, Valenti D, et al. (1988) Prenatal management of 746 pregnancies at risk for congenital toxoplasmosis. N Engl J Med 318(5):271-5.

31. Hohlfeld P, Daffos F, Thulliez P, Aufrant C, Couvreur J, MacAleese J, et al. (1989) Fetal toxoplasmosis: outcome of pregnancy and infant follow-up after in utero treatment . J Pediatr. 115(5 Pt 1):765-9.

32. Taglioretti A, Vucetich A, Agostoni G, Ravizza M, Meroni V, Semprini AG, et al. (1989) Prenatal diagnosis of congenital toxoplasmosis. Ann Ostet Ginecol Med Perinat. 110(1):49-54.

33. Couvreur J, Thulliez P, Daffos F, Aufrant C, Bompard Y, Gesquière A, et al. (1993) In utero treatment of toxoplasmic fetopathy with the combination pyrimethamine-sulfadiazine. Fetal Diagn Ther8(1):45-50.

34. Berrebi A, Kobuch WE, Bessieres MH, Bloom MC, Rolland M, Sarramon MF, et al. (1994) Termination of pregnancy for maternal toxoplasmosis. Lancet 344(8914):36-9.

35. Bessières MH, Berrebi A, Rolland M, Bloom MC, Roques C, Cassaing S, et al. (2001) Neonatal screening for congenital toxoplasmosis in a cohort of 165 women infected during pregnancy and influence of in utero treatment on the results of neonatal tests. Eur J Obstet Gynecol Reprod Biol 94(1):37-45.

36. Gilbert R, Gras L; European Multicentre Study on Congenital Toxoplasmosis. (2003) Effect of timing and type of treatment on the risk of mother to child transmission of Toxoplasma gondii. BJOG110(2):112-20.

37. Bessières MH, Berrebi A, Cassaing S, Fillaux J, Cambus JP, Berry A, et al. (2009) Diagnosis of congenital toxoplasmosis: prenatal and neonatal evaluation of methods used in Toulouse University Hospital and incidence of congenital toxoplasmosis. Mem Inst Oswaldo Cruz 104(2):389-92.

38. Valentini P , Annunziata ML, Angelone DF, Masini L, De Santis M, Testa A, et al. (2009) Role of spiramycin/cotrimoxazole association in the mother-to-child transmission of toxoplasmosis infection in pregnancy. Eur J Clin Microbiol Infect Dis 28(3):297-300.

39. Hotop A , Hlobil H, Gross U. (2012) Efficacy of rapid treatment initiation following primary Toxoplasma gondii infection during pregnancy. Clin Infect Dis 54(11):1545-52.

40. Saba J, Morlat P, Raffi F, Hazebroucq V, Joly V, Leport C, et al. (1993) Pyrimethamine plus azithromycin for treatment of acute toxoplasmic encephalitis in patients with AIDS. Eur J Clin Microbiol Infect Dis12(11):853-6.

41. Torres RA, Weinberg W, Stansell J, Leoung G, Kovacs J, Rogers M, et al. (1997) Atovaquone for Salvage Treatment and Suppression of Toxoplasmic Encephalitis in Patients with AIDS. Clinical Infectious Diseases 24:422-9.
